# Supplementary material for: Association between oral microbiome and seven types of cancers in East Asian population: a two-sample Mendelian randomization analysis
Source: Front Mol Biosci. 2023 Nov 21;10:1327893. doi: 10.3389/fmolb.2023.1327893 (PMC10702768; doi:10.3389/fmolb.2023.1327893)
Supplement: Supplementary file 1 [file DataSheet1.ZIP › Supplementary Materials/MR plots of saliva/saliva═╝/Prostate cancer/pheno.2370_to_prostate cancer_leave_one_out.pdf]

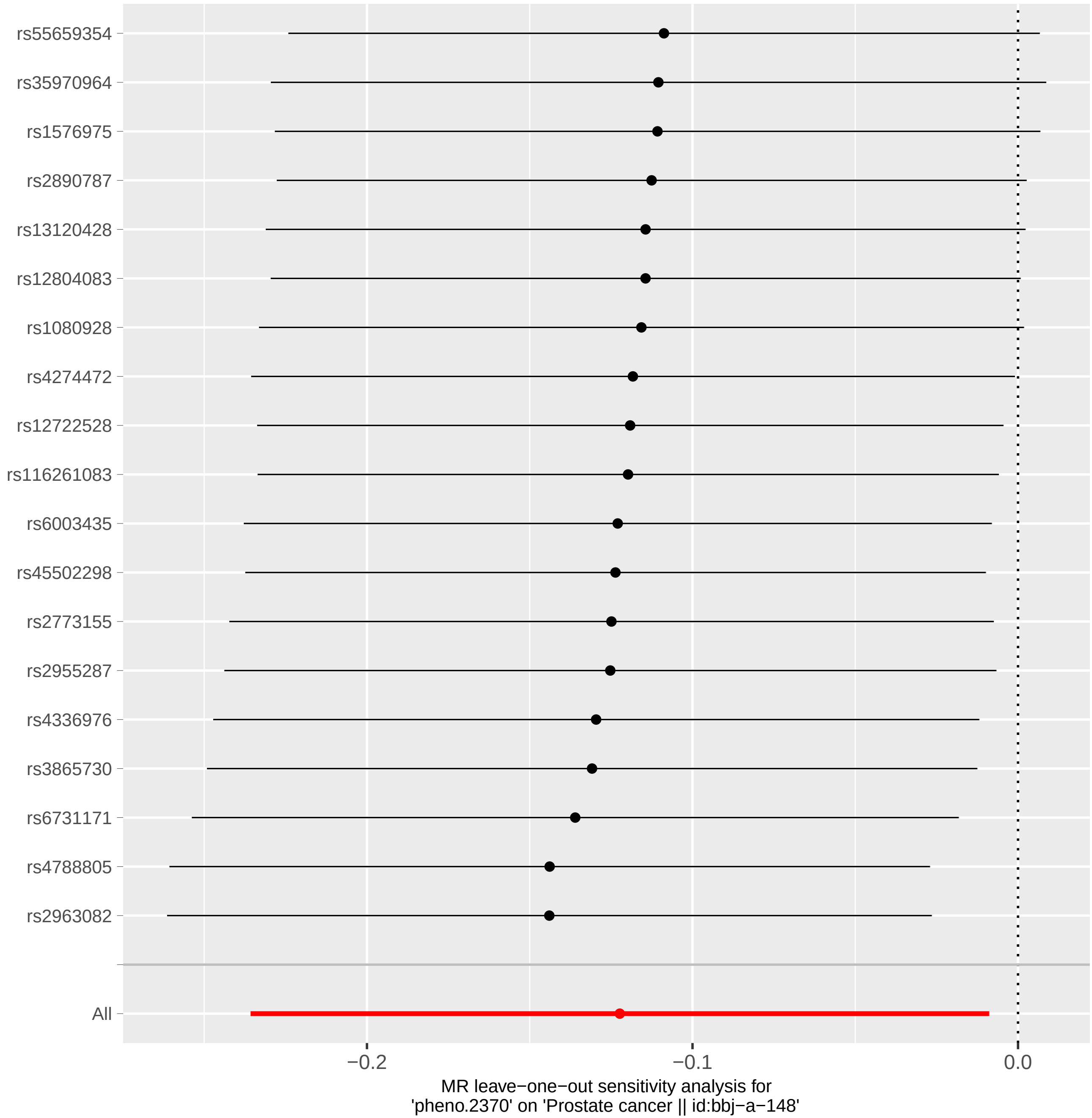
rs55659354
rs35970964
rs1576975
rs2890787
rs13120428
rs12804083
rs1080928
rs4274472
rs12722528
rs116261083
rs6003435
rs45502298
rs2773155
rs2955287
rs4336976
rs3865730
rs6731171
rs4788805
rs2963082
All
−0.2
−0.1
0.0
MR leave−one−out sensitivity analysis for
'pheno.2370' on 'Prostate cancer || id:bbj−a−148'
